# etymologia: *Shigella* [shĭ-gel′ə]

**DOI:** 10.3201/eid1503.999999

**Published:** 2009-03

Genus of gram-negative bacteria in the family *Enterobacteriaceae*, named for Japanese bacteriologist Koyoshi Shiga (1871–1957). In 1897, Japan experienced a severe dysentery epidemic; >91,000 cases were reported, and the case-fatality rate was >20%. Dr Shiga isolated the etiologic agent from patient stool samples: a bacillus, later called *Shigella dysenteriae*. He went on to describe the toxins that the organism produces; one that causes serious complications during infections is now known as Shiga toxin.

